# Recto-Vaginal Fistula

**Published:** 1895-11

**Authors:** A. Jasme

**Affiliations:** Savannah, Ga.


### RECTO-VAGINAL FISTULA.

By A. JASME, V.S.,
SAVANNAH, GA.

THE patient, a mare, had been owned for more than one year by present owner, during which time balls of fecal matter were excreted through vagina. The mare has during this time remained in good physical condition.

An examination revealed a recto-vaginal fistulous opening about four inches from the external orifice, and strongly resembling the anal orifice in appearance. Its size and shape seemed to be controlled by a sphincter, which would bear considerable dilatation.

I would like to know if this is congenital or if it has been caused by injury during labor? The anatomical aspect would favor the former opinion.

[A particularly interesting case of this character, with unique and successful surgical treatment, was reported by Prof. C. P.

Lyman, and a number of others somewhat similar by Dr. Youngbird, of Minnesota, at the recent annual meeting of the U. S. V. M. A. at Des Moines, Iowa.—EDITOR.]

